# Pennsylvania

**Published:** 1897-08

**Authors:** 


*Pennsylvania.*—An Act to Provide for the Investigation of the Diseases of Domestic Animals and making an appropriation therefor:

SECTION 1. Be it enacted by the Senate and House of Representatives of the Commonwealth of Pennsylvania in General Assembly met, and it is hereby enacted by the authority of the same, That the sum of fifteen thousand dollars, or so much thereof as may be neces-

**At Nashville, in September, will be the most important gathering to consider tuberculosis, ever held on American soil.**

sary, is hereby appropriated for the purpose of conducting investigations concerning the causes, nature, treatment, and prevention of the diseases of the domestic animals of the Commonwealth of Pennsylvania, with the object of discovering new facts which may be applied advantageously and profitably by the owners of live-stock and those engaged in the care, use, and rearing of animals.

**Experiment-station work, and how it can best accomplish its aim and purposes, will be considered.**

SEC. 2. That such investigations shall be made by and under the direction of the State Live-stock Sanitary Board, and the said board is hereby authorized to provide for and conduct such work of investigation as may be necessary to discover the most efficient, economical, and practical means of avoiding and suppressing the diseases of the domestic animals of this Commonwealth; and such work and the practical deductions therefrom shall, upon the order of the Secretary of Agriculture, be published as a part of the annual report of the Department of Agriculture, or as bulletins from said department.

SEC. 3. That all necessary expenses under the provisions of this act shall, after approval in writing by the Governor and the Secretary of Agriculture, be paid by the State Treasurer upon the warrant of the Auditor-General in the manner now provided by law.

SEC. 4. That this act shall take effect June first, one thousand eight hundred and ninety-seven.

The Horseshoers' Bill in this State was defeated on third reading in the House in June. The shoers will try again next session, when under more favorable circumstances it is expected that they will secure passage of their measure.

The Hamilton Good Roads Bill has passed the State Legislature, was signed by the Governor, and will be a long step in the right direction in promoting in the Keystone State the interests of horse-breeding, encourage more driving, and in every way contribute to the betterment of the equine industry.

